# Understanding the Flame Retardant Mechanism of Intumescent Flame Retardant on Improving the Fire Safety of Rigid Polyurethane Foam

**DOI:** 10.3390/polym14224904

**Published:** 2022-11-14

**Authors:** Seung Hun Lee, Seul Gi Lee, Jun Seo Lee, Byung Chol Ma

**Affiliations:** School of Chemical Engineering, Chonnam National University, Gwangju 61186, Korea

**Keywords:** RPU, flame retardancy, IFR, CCT

## Abstract

Combinations of multiple inorganic fillers have emerged as viable synergistic agents for boosting the flame retardancy of intumescent flame retardant (IFR) polymer materials. However, few studies on the effect of multiple inorganic fillers on the flame retardant behavior of rigid polyurethane (RPU) foam have been carried out. In this paper, a flame retardant combination of aluminum hydroxide (ATH) and traditional flame retardants ammonium polyphosphate (APP), pentaerythritol (PER), melamine cyanurate (MC), calcium carbonate (CC), and expandable graphite (EG) was incorporated into RPU foam to investigate the synergistic effects of the combination of multiple IFR materials on the thermal stability and fire resistance of RPU foam. Scanning electron microscopy (SEM) and thermogravimetric analysis (TGA) revealed that 8 parts per hundred polyols by weight (php) filler concentrations were compatible with RPU foam and yielded an increased amount of char residue compared to the rest of the RPU samples. The flame retardancy of multiple fillers on intumescent flame retardant RPU foam was also investigated using cone calorimeter (CCTs) and limiting oxygen index (LOI) tests, which showed that RPU/IFR1 (APP/PER/MC/EG/CC/ATH) had the best flame retardant performance, with a low peak heat release rate (PHRR) of 82.12 kW/m^2^, total heat release rate (THR) of 15.15 MJ/m^2^, and high LOI value of 36%. Furthermore, char residue analysis revealed that the use of multiple fillers contributed to the generation of more intact and homogeneous char after combustion, which led to reduced decomposition of the RPU foam and hindered heat transfer between the gas and condensed phases.

## 1. Introduction

Nowadays, rigid polyurethane (RPU) foam is widely used in the construction and packaging industries because it has excellent thermal insulation and noise reduction properties, moisture resistance, strong chemical resistance, and is light weight and low cost [1,2,3]. However, RPU foam decomposes quickly and produces a substantial amount of tiny molecular fragments in the gaseous phase due to its weak covalent bonding and high concentration of soft segments in the polyethylene chain [4,5]. Due to its poor flame retardancy, fire safety is an immense challenge when using RPU foam as a building material. In order to assure safety, researchers have been trying to improve the flame retardancy of RPU foam. Previously, RPU foam flame retardation was achieved by incorporating flame retardants into an RPU matrix (either physically or chemically) or by applying a functional layer via surface treatment. Physically combining additive-type flame retardants is a common method used to prepare flame retardant RPU foam. Currently, halogen-free flame retardants are widely used as flame retardant additives [6,7,8,9,10], including intumescent flame retardants (IFRs) such as ammonium polyphosphate (APP) as an acid source, pentaerythritol (PER) as a carbon source, and melamine cyanurate (MC) as a blowing agent. Expandable graphite (EG) is widely used as a halogen-free additive [11]. When exposed to heat, an intumescent coating swells up and forms a char layer between the gas and condensed phases. These compact char layers act as physical barriers that reduce heat transfer to the substrate layer. Though IFRs have several advantages, such as excellent fire protection, low smoke levels, and low toxicity, their weak flame retardancy and moderate thermal stability limit their commercial utility [12,13,14,15]. A significant loading of flame retardant additives is required to achieve satisfactory performance but also limits the mechanical and thermal properties of RPU foam [16,17,18].

On the other hand, the use of inorganic fillers can improve flame retardancy because they can change the chemical and physical properties of intumescent char throughout the burning process [19]. Currently used inorganic fillers include silica, montmorillonite, talc, and calcium carbonate, among which calcium carbonate is the most widely used inorganic filler due to its low cost, high thermal stability, and availability [20,21,22,23,24,25]. In a recent study, it was observed that CaCO_3_ had a positive influence on the fire retardant characteristics of intumescent composition. During combustion, CaCO_3_ reacts with phosphate species, thus leading to the formation of thermally stable complexes and improving flame retardant performance [26]. However, flame retardant requirements cannot be met with the addition of a single inorganic filler. It is widely known that the combination of various fillers can improve the performance of flame retardants. Currently, aluminum hydroxide (ATH) is considered to be a classic inorganic flame retardant material due to its high stability, low toxicity, and long-lasting flame retardant effect. During combustion, ATH undergoes endothermic dehydration, releasing water into the gas phase while forming a thermally stable ceramic-like protective layer on the surface of the polymer [27,28,29,30]. This protective layer acts as a barrier that boosts flame retardant performance.

In this work, multiple fillers, CaCO_3_ and ATH, were used as synergists with the APP/PER/MC system, as the main flame retardant in an RPU-based flame retardant system. Furthermore, the influence of multiple filler concentrations (5, 8, and 10 php) on the RPU/IFR foam was investigated. The thermal degradation behavior and flame retardant performance of the RPU foam samples were investigated using thermogravimetric analysis (TGA) and cone calorimeter tests (CCTs). In addition, the char residue was analyzed using scanning electron microscopy–energy dispersive X-ray spectroscopy (SEM–EDS) and Raman spectroscopy to understand the flame retardant mechanism.

## 2. Materials and Methods

### 2.1. Materials

The primary components of this investigation were polyols, isocyanates, catalysts, blowing agents, ammonium polyphosphate (APP), pentaerythritol (PER), melamine cyanurate (MC), calcium carbonate (CaCO_3_), aluminum hydroxide (ATH), and expandable graphite (EG). Table 1 contains information regarding the suppliers of these components, as well as their specifications.

### 2.2. Preparation of Polyurethane Foam

The flame retardant RPU foam samples were prepared using the one-pot and free-rise method. The polyol (Stepanol PS- 3152, polyester polyol), catalysts (Dabco K-15, potassium-octoate), surfactant (polysiloxane silicon), and IFR (APP/PER/MC/EG/CC/ATH) were well-mixed in a 1 L beaker. Next, MDI was added into the beaker with vigorous stirring for 10 s. The mixture was immediately poured into an open mold (300 × 200 × 150 mm^3^) to produce free-rise foam. The foam was cured under ambient conditions. The formulations of the RPU foam are shown in Table 2.

### 2.3. Characterization

Surface morphology: The surface morphological images were recorded using a scanning electron microscope (SEM; S-4700, Hitachi). Energy dispersive X-ray spectroscopy (EDS) was used to analyze the elemental content of samples, which was carried out on the same SEM using an X-Max20 X-ray probe.

Thermal Analysis: Thermal gravimetric analysis (TGA) of the neat RPU and flame retardant RPU samples was performed using a heating rate of 10 °C/min from RT up to 800 °C under a nitrogen atmosphere using TA Instruments SDTA 851E.

Density: The density of the neat RPU and flame retardant RPU samples was measured according to ASTM D1622. The size of each sample was 20 × 20 × 2.5 mm^3^ and the average value of at least 3 samples was obtained.

Thermal conductivity: Thermal conductivity was measured using the transient plane source technique with a Hot Disk (FOX 200, New Castle, PA, USA) instrument at room temperature according to ASTM C518-91.

Cone Calorimetry Test (CCT): An FTT standard cone calorimeter (Fire Testing Technology Ltd., East Grinstead, UK) was used to evaluate the flame retardance of the prepared RPU foam samples according to ISO 5660 under an external heat flux of 50 kW/m^2^ with a specimen dimension of 100 × 100 × 50 mm^3^.

Limiting oxygen index (LOI) test: The limiting oxygen index (LOI) test was carried out using an FTT (Fire Testing Technology Ltd., East Grinstead, UK) Dynisco LOI instrument in accordance with ASTM D2863–17 with a sample dimension of 100 × 10 × 10 mm^3^.

Raman analysis: The micro-Raman spectrum of the char residue was recorded in the spectral range of 100–3000 cm^−1^ using a micro-Raman spectrometer (inVia Reflex UV Raman microscope (Renishaw, UK) at KBSI (Gwangju-Center, Korea). A He-Ne laser source was employed with an excitation wavelength of 633 nm and resolution of 1 cm^−1^ at 15 mW laser power.

## 3. Results and Discussion

### 3.1. Surface Morphology

**Figure 1:**
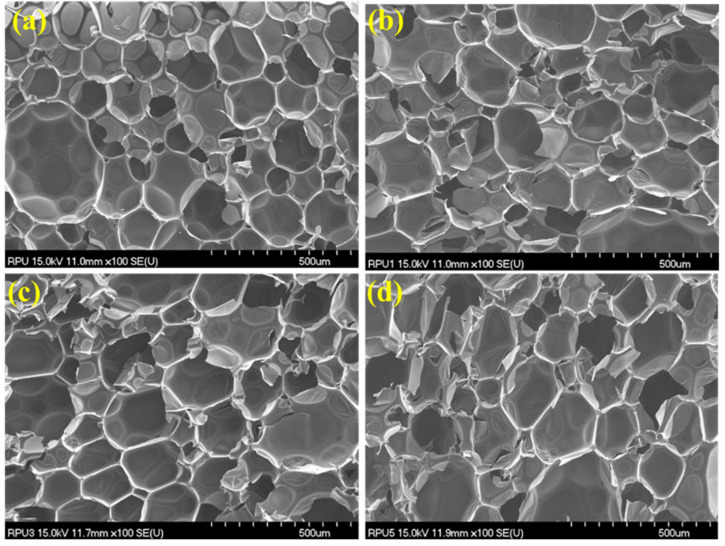
SEM images of the neat RPU and flame retardant RPU foam samples: (**a**) neat RPU, (**b**) RPU/IFR0, (**c**) RPU/IFR1, and (**d**) RPU/IFR2.

The morphology and microstructure of the pure RPU foams and flame retardant RPU composite foams were characterized using SEM, and the images are shown in Figure 1. It can be seen that the pure RPU foam exhibited a typical closed-cellular polyhedron structure. No collapse or collision was observed in the cell system, and the average cell diameter was approximately 220 μm (Figure 1a). The surfaces of the walls of the RPU foam struts were smooth and had a relatively uniform distribution. The flame retardant RPU foams also exhibited closed-cell structures with near-spherical cells (Figure 1b–d). Moreover, during the preparation of the SEM samples, most of the flame retardant was separated from the center portion, indicating that the bonding strength between the flame retardant and the PU matrix was quite high, and the flame retardant could not be readily peeled off the matrix. Furthermore, the cell size of the flame retardant RPU foams gradually increased from 250 to 266 μm and then to 279 μm.

### 3.2. Thermogravimetric Analysis

**Figure 2:**
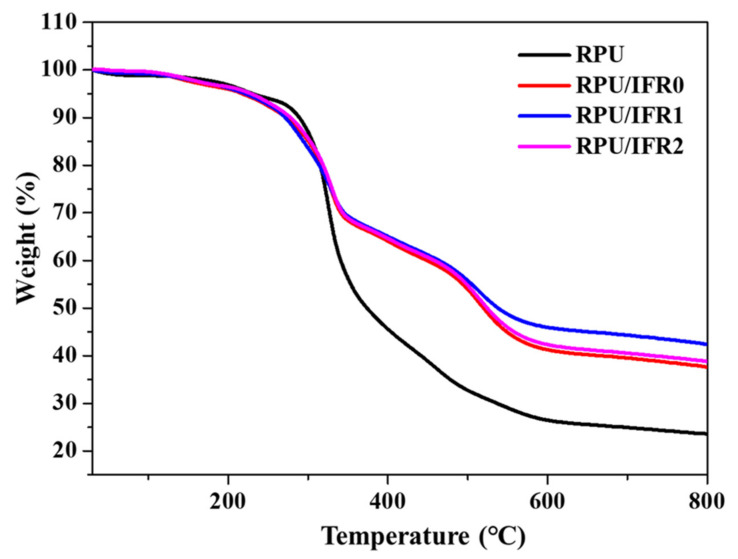
TGA curves of the neat RPU and flame retardant RPU foam samples under a nitrogen atmosphere at a heating rate of 10 °C/min.

The thermal behavior of the neat RPU and flame retardant RPU foam samples was investigated via thermogravimetric analysis (TGA). Figure 2 shows the TGA curve of the various RPU foams under N_2_ atmosphere. It can be seen from Figure 2 that the neat RPU foam degradation process consisted of two major weight loss steps. The initial degradation was observed between 150 and 250 °C, with an 8% weight loss. This step was attributed to the evaporation of small molecules of unreacted isocyanate monomers [31]. In the second step, a major 67% weight loss was observed between approximately 250 and 650 °C, corresponding to the dissociation of urethane bonds in the hard segment. Following decomposition, the polyol segments turned into aliphatic ether alcohols, olefins, and CO_2_. At higher temperatures of ~500 °C, the degradation process led to the generation of volatile products such as CO_2_, HCN, and NO_2_ from other products derived from the isocyanate group (such as amines or benzene alkyl) [32]. The decomposition temperatures of the flame retardant RPU foams were less than that of the neat RPU foam, and the final char residue weight varied among different flame retardant combinations. These results indicated that the flame retardant additives interacted to form an intumescent char layer that served as protective barrier to prevent further decomposition of the sample. For the RPU/IFR0 sample, major weight loss was observed between 180 and 580 °C, which was related to the decomposition of EG and interaction between flame retardant additives that led to an enhanced char residue at 800 °C. In detail, when the temperature was higher than 190 °C, EG started to decompose and release SO_2_ gas while APP also started to decompose and release NH_3_ and H_2_O to form polyphosphoric acid [33]. As the temperature increased, esterification between phosphoric acid and PER occurred. Furthermore, an NH_2_ group from MC interacted with an NCO group of MDI and generated a polymeric network. Rising temperatures led to the release of nonflammable gases, which diluted the oxygen concentration, and swelled the precursor of the intumescent char [34]. As the temperature increased, calcium carbonate reacted with polyphosphoric acid and produced thermally stable char.

For the RPU/IFR1 sample, ATH was found to play a key role in enhancing the flame retardant performance of the intumescent flame retardant RPU foam. The TGA curve revealed a small weight loss in the 265–310 °C region. In this process, ATH underwent endothermic dehydration, releasing water and generating aluminum oxide. Furthermore, when the temperature exceeded 550 °C, polyphosphoric acid reacted with aluminum oxide and generated aluminum phosphate, thus leading to improved flame retardant performance and final char residual weight [35,36]. The residual weight percentages were 23.80% and 37.57% for the neat RPU and RPU/IFR0 foam samples, respectively, and 42.67% and 38.80% for the RPU/IFR1 and RPU/IFR2 samples, respectively.

Moreover, final residual weight increases were observed at 800 °C, indicating the existence of synergism between IFR and ATH that led to the generation of thermally stable intumescent char. This char could serve as an effectively physical barrier layer during the combustion of RPU foam [37,38]. In particular, the RPU/IFR1 sample exhibited the highest residual weight percentage among the samples, indicating that this combination had the best synergistic effect on char-forming ability.

### 3.3. Cone Calorimeter Tests

**Figure 3:**
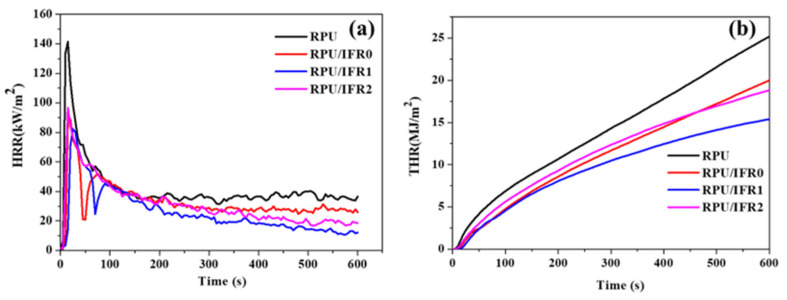
Cone calorimeter curves of neat RPU, RPU/IFR0, RPU/IFR1, and RPU/IFR2 foam samples: (**a**) PHRR and (**b**) THR.

The cone calorimeter test is widely utilized by fire safety engineers and scientists for the quantitative examination of material flammability. It continues to be one of the most valuable bench-scale tests aimed at imitating real-world fire conditions [39]. The peak heat release rate (PHRR), total heat release (THR), maximum average rate of heat emission (MARHE), and average effective heat of combustion (Av-EHC) are well-known as crucial factors for evaluating flammability behavior and could be utilized to express fire intensity and fire spread rate. Figure 3a,b depicts the PHRR and THR curves of the neat RPU and flame retardant RPU foam samples, and detailed data are shown in Table 3. In the neat RPU foam, the low thermal inertia of the insulating foam and porous nature of the RPU foam increased the contact area between the matrix and oxygen, which led to the neat RPU foam quickly burning following ignition, with high PHRR and THR values of 140 kW/m^2^ and 25.06 MJ/m^2^, respectively. On the other hand, incorporating flame retardants into the RPU foam led to obvious reductions in PHRR and THR. The RPU/IFR0 sample exhibited drastically reduced PHRR and THR values of 88.97 kW/m^2^ and 19.68 MJ/m^2^, respectively. These results demonstrated that the flame retardant materials generated intumescent char during the combustion process, which led to the increased flame retardancy of the RPU foam. Furthermore, the addition of ATH seemed to significantly reduce the PHRR and THR values to 82.12 kW/m^2^ and 15.15 MJ/m^2^, respectively, in the RPU/IFR1 sample. However, the increased ATH content in the RPU/IFR2 sample led to increased PHRR and THR values of 96.05 and 18.69, respectively. An excessive content of ATH might have consumed too much APP, which affected crosslinking between APP and PER and therefore limited the synergistic effect of the intumescent materials, thus reducing the flame retardant performance. These results confirmed that a moderate amount of multiple filler-based flame retardant material had the potential to improve the flame retardancy of RPU foam.

The effective heat of combustion (EHC), which is derived from the ratio between the heat release rate (HRR) and mass loss rate, refers to the degree of burning of fuels or flammable species from the matrix pyrolysis during combustion and is useful for examining the mechanism of action of flame retardants [40]. As shown in Table 4, the average EHC values (av-EHC) of the RPU samples decreased as the filler content increased, which demonstrated that the content of combustion components in the flame retardant RPU samples was reduced. The risk of a fire spreading can be expressed using the term MARHE [41]. Although 90.0 kW/m^2^ was found to be the MARHE value for the neat RPU foam sample, the MARHE values greatly decreased to 53.7 and 48.9 for the RPU/IFR0 and RPU/IFR1 samples, respectively. However, for the RPU/IFR2 sample, the MARHE value increased to 60.7 as compared to the other flame retardant RPU samples.

The RPU/IFR1 sample showed the lowest PHRR, THR, av-EHC, and MARHE values among the RPU samples, suggesting that the incorporation of a moderate content of ATH into the intumescent system enhanced the flame retardant performance of the RPU foam during combustion. During the combustion process, ATH was converted into aluminum oxide while releasing water. Furthermore, at higher temperatures, aluminum oxide and CaCO_3_ reacted with polyphosphoric acid and generated a thermally stable layer of aluminum orthophosphate, aluminum metaphosphate, calcium phosphate, and calcium metaphosphate on the surface of the coating [42,43], thus leading to improved flame retardancy.

### 3.4. Limiting Oxygen Index (LOI) Tests

The limiting oxygen index (LOI) test is the most commonly used method to determine the flame retardant performance of RPU foam, and the results can be used to supply pivotal evidence for judging the application value [44]. Detailed data obtained from the LOI tests of the RPU samples are shown in Table 4. The neat RPU foam was shown to be highly flammable under atmospheric conditions, with a significantly low LOI value of 22%. However, it can be seen that the LOI values of the flame retardant RPU foams were higher than that of the neat RPU foam material. For the RPU/IFR0 sample, the LOI value increased to 34%; these results may be attributed to the fact that during combustion, flame retardant materials generate an intumescent char layer, which acts as a barrier that leads to improvements in the flame retardancy of the RPU foam. Furthermore, the LOI value improved with the incorporation of ATH into the flame retardant system. The RPU/IFR1 and RPU/IFR2 samples displayed LOI values of 36% and 35%, respectively. The LOI value of the RPU/IFR2 sample was lower than that of the RPU/IFR1 sample, indicating that an excessive amount of ATH diminished the synergy between ATH and the intumescent flame retardant additives. We observed that the incorporation of a moderate amount of ATH in the intumescent flame retardant system led to more compact char layers.

### 3.5. Combustion Behavior and Char Residue Analysis

**Figure 4:**
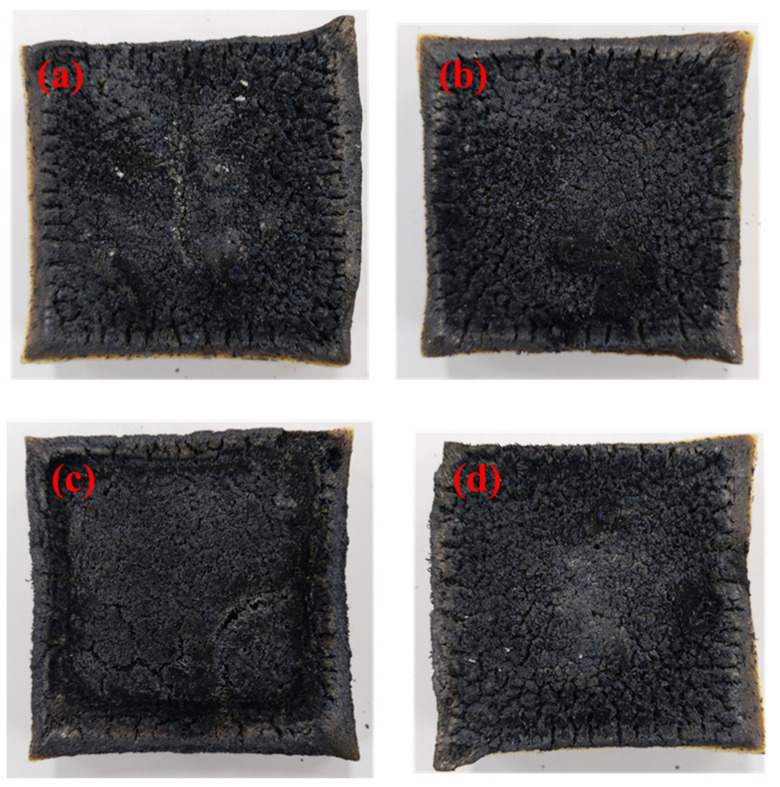
Digital photographs of combustion phenomenon of (**a**) neat RPU, (**b**) RPU/IFR0, (**c**) RPU/IFR1, and (**d**) RPU/IFR2.

Cone calorimetry tests were carried out in order to simulate the combustion behavior of a real fire hazard, and digital photos of the neat RPU and flame retardant RPU foams following the cone calorimeter test are shown in Figure 4. It can be observed in the digital photograph shown in Figure 4a that the neat RPU foam produced a discontinuous and broken residual char layer after combustion. In addition, we observed that the incorporation of flame retardant materials into the RPU foam produced intumescent char after the combustion process. Figure 4b shows that the RPU/IFR0 sample had a reduced amount of cracks on the surface of the residual char compared with that of the neat RPU foam after combustion. In contrast, Figure 4c shows that the RPU/IFR1 sample produced a more compact char layer. This compact char acted as a barrier layer during the combustion process, which resulted in the lowest PHRR and THR values of the samples. However, Figure 4d shows that the RPU/IFR2 sample produced a more discontinuous and broken char layer relative to the RPU/IFR1 sample. Furthermore, the weak intactness provided limited resistance to heat, which ultimately reduced its flame retardant behavior.

### 3.6. Char Residue Analysis

**Figure 5:**
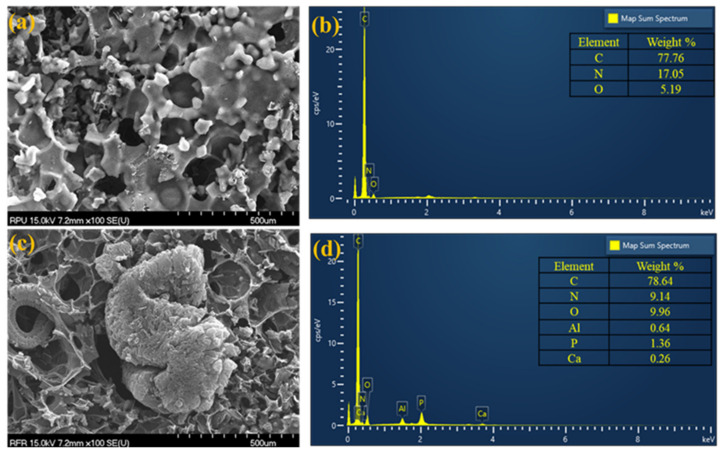
SEM images (**a**,**c**) and corresponding EDX spectra (**b**,**d**) of the char residues of the neat RPU (**a**,**b**) and RPU/IFR1 foam samples (**c**,**d**).

After the CCTs, the char residues were examined using scanning electron microscopy (SEM) to investigate the specific mechanism of the flame retardant RPU foam. Figure 5a depicts an SEM micrograph of the residual char of the neat RPU sample, revealing that the cells were significantly damaged and there was a significant amount of slag on the cell surface. The damaged cells provided limited resistance to heat, which led to poor flame retardant performance during the combustion process. Further, the EDX analysis shown in Figure 5b indicated that the char residue of the neat RPU sample contained only C, N, and O elements. Furthermore, Figure 5c depicts the SEM micrograph of the residual char of the RPU/IFR1 sample, which exhibited a more compact and denser worm-like intumescent char layer than the other RPU samples. This compact intumescent char acted as an effective barrier layer during combustion. Moreover, the EDX spectrum of the char residue of the RPU/IFR1 sample in Figure 5d revealed the presence of C, N, and higher O content with additional P, Ca, and Al elements. These results indicated the presence of a thermally stable structure on the surface of the char, which led to the RPU/IFR1 sample showing improved fire resistance performance. Thus, the SEM–EDX results confirmed the beneficial effect of the multiple fillers with IFR in boosting the flame retardant performance of the RPU/IFR1 sample.

### 3.7. Raman Analysis

**Figure 6:**
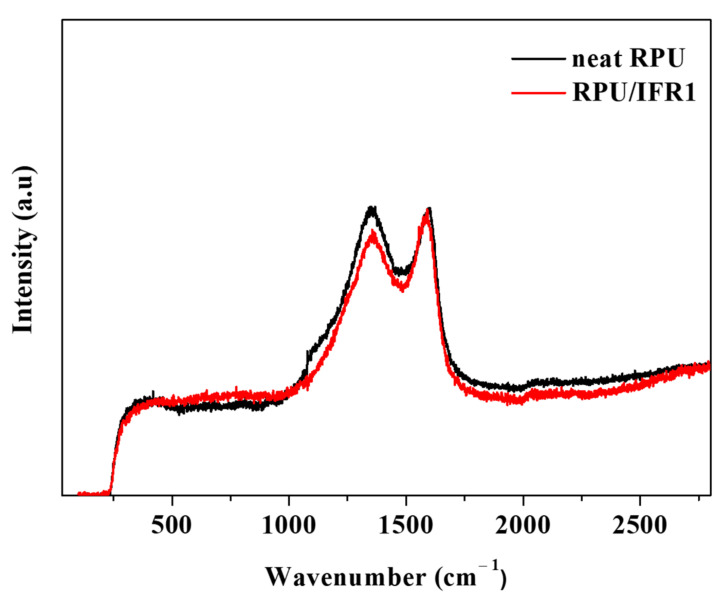
Raman spectra of the char residues collected from neat RPU and RPU/IFR1 foam samples after the CCTs.

In order to analyze the quality of the char and its various forms of carbonaceous materials (especially those generated during the combustion process), char residues of the neat RPU and flame retardant RPU/IFR1 foam samples were analyzed using Raman spectroscopy. It is well-known that the efficiency of a flame retardant material depends on both the quantity and quality of the char residues present. Figure 6 shows the Raman spectra of the char residues collected from the neat RPU and RPU/IFR1 foam samples after the CCTs. The Raman spectra of carbon signals typically exhibit a D band at 1360 cm^−1^ and a G band at 1580 cm^−1^; the D and G bands have different intensities, which is attributed to amorphous and graphitized carbon contents [45]. A lower I_D_/I_G_ ratio suggests a stable char structure with more graphitization, implying improved fame retardancy [46]. The calculated I_D_/I_G_ ratio of the RPU/IFR1 sample (0.97) was lower than that of the neat RPU foam (1.00), indicating a residue with a higher degree of graphitization.

### 3.8. Physical Properties of RPU Foam

The cell size, cell uniformity, and strength of the cell wall all play roles in the thermal and mechanical strength of RPU foams [47]. However, the mechanical strength of RPU foams is generally determined by foam density and cell structure. In this study, the influence of multiple inorganic fillers with IFR on RPU foams was tested, and the results are summarized in Table 4. The flame retardant RPU foams were denser than the neat RPU foam. The neat RPU foam exhibited a density of 61.1 kg/m^3^, which was increased to 64.2, 65.0, and 65.6 kg/m^3^ for RPU/IFR0, RPU/IFR1, and RPU/IFR2, respectively. This increased density may be attributed to the incorporation of combinations of multiple fillers, which led to increased viscosity.

Thermal conductivity is an important tool to evaluate the thermal insulation performance of polymeric materials. Data on the thermal conductivity of the neat RPU and flame retardant RPU foams are shown in Table 4. It can be seen that compared with the neat RPU foam, the thermal conductivity of the flame retardant RPU foams was drastically increased. As expected, the addition of multiple flame retardant additives significantly affected the pore size of the flame retardant RPU foam, and its thermal conductivity was affected by the cell structure. It can be seen that the thermal conductivity increased as cell size increased. We observed thermal conductivity values of 0.0163 W/m·k for the neat RPU foam, 0.0168 W/m·k for RPU/IFR0, 0.0169 W/m·k for RPU/IFR1, and 0.0170 W/m·k for RPU/IFR2. It was determined that the thermal conductivity of the flame retardant RPU foam increased while maintaining adequate thermal insulation properties.

## 4. Possible Flame Retardant Mechanism

**Figure 7:**
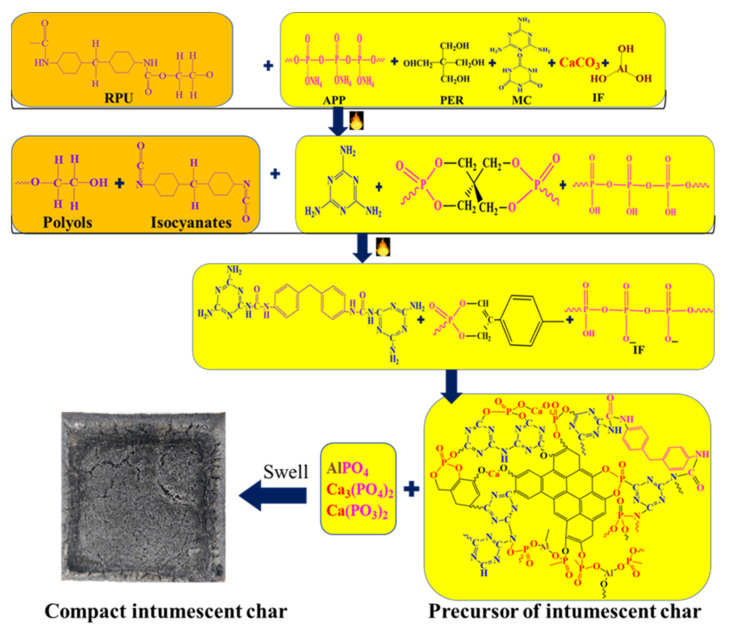
Possible flame retardant mechanism during the combustion process. Here, the inorganic filler (IF) contains CaCO_3_ and ATH.

Based on our analysis, the possible flame retardant mechanism of the RPU/IFR1 sample was formulated and is shown in Figure 7. When flame retardant RPU foam is ignited, EG begins to expand, which results in the formation of a “worm-like” intumescent char layer. During the expansion process, EG can absorb a considerable amount of heat, which results in the generation of a stable char layer. At the same time, ATH starts to decompose, releasing a water molecule and generating aluminum oxide. Meanwhile, APP decomposes to release water and ammonia and produce polyphosphoric acid. At this stage, the degradation process of polyurethane foam occurs. Furthermore, polyphosphoric acid reacts with PER to form an ester mixture. As the temperature rises, interactions between MC and MDI lead to the formation of a polymeric network that improves the barrier during combustion.

Increasing temperature leads to the generation of a precursor to intumescent char, accompanied by the release of gaseous products. Furthermore, at higher temperatures, polyphosphoric acid reacts with aluminum oxide and calcium carbonate to generate thermally stable calcium metaphosphate and aluminum phosphate, which positively affects the barrier during combustion and the anti-oxidation capacity of the char layer. Therefore, the use of multiple fillers with IFR in the condensed phase exerts a synergistic effect.

## 5. Conclusions

Flame retardant RPU foams were prepared by incorporating intumescent flame retardant materials with CC and ATH as inorganic fillers. A homogenous dispersion of multiple flame retardant materials in the PU matrix was achieved, which led to significantly improved flame retardancy of the RPU foam. In particular, the incorporation of a moderate amount of inorganic fillers achieved excellent flame retardant performance in RPU foam. The CCTs revealed that the PHRR value of the flame retardant RPU foam was reduced from 140 to 82.12 kW/m^2^ and the THR value was reduced from 25.06 to 15.15 MJ/m^2^. The TGA results showed that the decomposition temperature of the flame retardant RPU foam decreased but the char yield increased. Furthermore, in studying the char residues, we found that the incorporation of combinations of multiple fillers could generate a more dense and compact char, which acted as a protective barrier during the combustion process and led to increased flame retardant performance of the RPU foam.

## Figures and Tables

**Table 1:** Specifications of the materials used in this work.

Materials	Specification
Polyol	Stepanol PS-3152, polyester polyol, 315 mg/g Hydroxyl value, purchased from STEPAN, Anaheim, CA, USA
Isocyanate	4’4-Methylene diphenyl diisocyanate (MDI)
Catalyst	Dabco K-15, potassium-octoate obtained from EVONIC, Rheinfelden, Germany
Polysiloxane silicon	TEGOSTAB B-8462, surfactant, purchased from EVONIC, Rheinfelden, Germany
HC- Cyclopentane	Blowing agent from SK Geocentric
Ammonium polyphosphate (APP)	Flame retardant, acid source, purity > 98%, particle size d50 of ~8 μm, obtained from Samchun Pure Chemicals, Seoul, Korea
Pentaerythritol (PER)	Flame retardant, carbonizing agent, purity 98%, obtained from Samchun Pure Chemicals, Seoul, Korea
Melamine cyanurate (MC)	Flame retardant, blowing agent, purity 99%, particle size 1.8 μm, obtained from Samchun Pure Chemicals, Seoul, Korea
Expandable graphite (EG)	Flame retardant, purity 99%, particle size is 80% >50 mesh, expansion rate is over 350 cm^3^/g, pH 7.0, obtained from Samjung C&G, Ulsan, Korea
Calcium carbonate (CC)	Flame retardant filler, purity 98.5%, particle size 3.5 μm, obtained from Samchun Pure Chemicals, Seoul, Korea.
Aluminum hydroxide (ATH)	Flame retardant filler, purity 63% particle size ~6 μm, purchased from Daejung Chemicals, Gyeonggi, Korea and used without further purification.

**Table 2:** Preparative parameters of flame retardant RPU foam formulations.

Samples	Basic Composition (pphp)						Flame Retardant (php)				
	Polyol	Catalyst	Surfactant	Blowing Agent	MDI	APP	PER	MC	EG	CC	ATH
RPU	100	4.2	5.0	20.0	150	-	-	-	-	-	-
RPU/IFR0	100	4.2	5.0	20.0	150	15	5	5	15	5	-
RPU/IFR1	100	4.2	5.0	20.0	150	15	5	5	15	5	3
RPU/IFR2	100	4.2	5.0	20.0	150	15	5	5	15	5	5

pphp, part per 100 parts of polyol by weight (g); mixing ratio: ROH: RNCO = 1:1.5.

**Table 3:** The cone calorimeter test results for different RPU samples.

Sample Code	PHRR (kW/m^2^)	THR (MJ/m^2^)	Av-EHC (MJ/kg)	MARHE (kW/m^2^)
RPU	140	25.06	33.71	90.0
RPU/IFR0	88.97	19.68	11.02	53.7
RPU/IFR1	82.12	15.15	6.32	48.9
RPU/IFR2	96.05	18.69	9.40	60.7

**Table 4:** Physical properties and LOI test results of neat RPU and flame retardant RPU foam samples.

Sample	Density (kg/m^3^)	Thermal Conductivity (W/m·k)	LOI
Neat RPU	61.1	0.0163	22
RPU/IFR0	64.2	0.0168	34
RPU/IFR1	65.0	0.0169	36
RPU/IFR2	65.6	0.0170	35

## Data Availability

All data has been provided i within this manuscript.
